# Metabolites in Blood for Prediction of Bacteremic Sepsis in the Emergency Room

**DOI:** 10.1371/journal.pone.0147670

**Published:** 2016-01-22

**Authors:** Anna M. Kauppi, Alicia Edin, Ingrid Ziegler, Paula Mölling, Anders Sjöstedt, Åsa Gylfe, Kristoffer Strålin, Anders Johansson

**Affiliations:** 1 Department of Clinical Microbiology, Clinical Bacteriology, the Laboratory for Molecular Infection Medicine Sweden and Umeå Centre for Microbial Research, Umeå University, Umeå, Sweden; 2 Department of Infectious Diseases, Örebro University Hospital, Örebro, Sweden; 3 Department of Laboratory Medicine, Faculty of Medicine and Health, Örebro University, Örebro, Sweden; 4 Department of Infectious Diseases, Karolinska University Hospital, Stockholm, Sweden; University of Pittsburgh, UNITED STATES

## Abstract

A metabolomics approach for prediction of bacteremic sepsis in patients in the emergency room (ER) was investigated. In a prospective study, whole blood samples from 65 patients with bacteremic sepsis and 49 ER controls were compared. The blood samples were analyzed using gas chromatography coupled to time-of-flight mass spectrometry. Multivariate and logistic regression modeling using metabolites identified by chromatography or using conventional laboratory parameters and clinical scores of infection were employed. A predictive model of bacteremic sepsis with 107 metabolites was developed and validated. The number of metabolites was reduced stepwise until identifying a set of 6 predictive metabolites. A 6-metabolite predictive logistic regression model showed a sensitivity of 0.91(95% CI 0.69–0.99) and a specificity 0.84 (95% CI 0.58–0.94) with an AUC of 0.93 (95% CI 0.89–1.01). Myristic acid was the single most predictive metabolite, with a sensitivity of 1.00 (95% CI 0.85–1.00) and specificity of 0.95 (95% CI 0.74–0.99), and performed better than various combinations of conventional laboratory and clinical parameters. We found that a metabolomics approach for analysis of acute blood samples was useful for identification of patients with bacteremic sepsis. Metabolomics should be further evaluated as a new tool for infection diagnostics.

## Introduction

The World Economic Forum has identified antibiotic resistance as one of the greatest risks of human health [1]. As antibiotic resistance is emerging, antibiotic choices that were considered to be reliable a decade ago for treating bacteremic sepsis may be uncertain treatment options today. The number of excess deaths among patients with bacteremia in Europe, attributable to antibiotic resistance exceeded 8,000 in year 2007 for *Staphylococcus aureus* and *Escherichia coli* infections, and trajectories for 2015 suggest 17,000 fatalities [2]. Reduction of unnecessary antibiotic use has been identified as one of the most important issues in order to stop the emergence of antibiotic resistance [3]. There is an urgent need for diagnostic tools that can support antibiotic decisions, so antibiotics can be given to patients who need them, but can be withheld in patients who do not.

Metabolomics, the comprehensive analysis of metabolites is a rapidly developing diagnostic tool for metabolic classification of individuals. The metabolome is smaller than the complex proteome or transcriptome of the human body, and thus, more amenable to a comprehensive analysis. Moreover, the metabolome is predictive of the phenotype and responds directly to genetic changes, disease or external factors. [4, 5] It has been demonstrated that pneumococcal pneumonia could be discriminated from other types of pneumonia [6] and that global metabolomic profile in plasma broadly differs between survivors and non-survivors of community acquired pneumonia and sepsis [7, 8]. In experimentally infected mice, metabolic profiling could distinguish effective from ineffective antimicrobial treatments of antibiotic resistant *S*. *aureus* [9].

In this study we analyzed blood samples from patients with suspected sepsis by GC-TOF-MS. We found that the metabolites identified performed well in diagnosis of bacteremic sepsis.

## Methods

### Patient samples

In a prospective study from October 2007 to September 2008 we included 1,093 consecutive adult patients, who were subjected to blood culturing in the Emergency room (ER) or within 4 hours after admission to the Department of Infectious Diseases, Örebro University Hospital, Sweden [10]. Whole blood was collected in sterile EDTA tubes (BD Vacutainer™ K3E 15%, Becton, Dickinson and Company, Plymouth, UK) through the same venepuncture from which blood samples for blood culture were taken. The whole blood was kept for a maximum of 4 h at room temperature or up to 3 days at 4°C. The blood was aliquoted into Cryo tubes before frozen at −80°C. For the present study patient samples with confirmed bacteremic sepsis positive for *E*. *coli*, *S*. *aureus*, *Klebsiella pneumoniae*, *Streptococcus pneumoniae*, or *Streptococcus pyogenes* were included. Samples that had been freeze thaw cycled were excluded rendering inclusion of 65 out of a total of 138 blood culture positive patients. Forty-nine ER control samples were included. The ER controls were patients with: 1) negative blood culture and a laboratory confirmed diagnosis explaining a clinical suspicion of bacteremic sepsis at admission (viral infection, reactive arthritis, borrelia, or tuberculosis), and 2) similar age and sex distribution as for the bacteremic sepsis samples. All samples were thawed once at room temperature and 100 μl of whole blood was aliquoted into Eppendorf tubes (Sarstedt) and frozen at −80°C until extraction. A retrospective chart review was performed to evaluate the severity of illness [11]. The patient's clinical condition was classified by using the criteria for systemic inflammatory response syndrome (SIRS), sepsis and septic shock published by the American College of Chest Physicians/Society of Critical Care Medicine [12]. The study subjects provided their written informed consent and the regional ethics committee in Uppsala, Sweden approved the study (Dnr. 2007/071).

### Extraction, derivatization and GC-TOF-MS analysis

Samples were divided into batches and the order of samples was randomized within batches. Each batch included similar variations in age, gender and infection types. Whole blood (100μl) was thawed at ambient temperature for 15 min and thereafter kept on ice. In brief, extraction was performed according to a published method [13] with the modification of using 900μl MeOH/CHCl_3_/H_2_O (60:20:20 v/v) as extraction mixture. Samples were extracted in a bead mill (MM400, Retsch GmbH, Haan, Germany) for 2 min at 30 Hz, followed by two hours incubation at 4°C before centrifugation at 14,000 rpm for 10 min at 4°C. 200 μl supernatant was transferred to GC/MS vials and dried in a speedvac (miVac, Quattro concentrator, Barnstead Genevac, Ipswich, UK) until dryness (typically 2–3 hours) and thereafter stored at -80°C until derivatization. Quality control samples consisting of pooled aliquots of whole blood samples for all patient and control material were included in every batch.

The samples were evaporated for 20 min to ensure complete dryness before derivatization. Methoxymation was carried out at 75°C for one hour. The samples were trimethylsilylated by addition of 40 μl N-methyl-N-trimethylsilyl-trifluoroacetamide +1% Trimethylchlorosilane followed by 30 min incubation at 75°C. Just before analysis, 40 μl heptane including methylstearate (15 ng/μl) was added. GC-TOF-MS analysis was performed in accordance with a previously published method [14] with slight modifications. One microliter of the derivatized sample was injected splitless by an CTC Combi Pal Xt Duo (CTC Analytics AG, Switzerland) auto-sampler/robot into an Agilent 7890A gas chromatograph equipped with a 30 m×0.25 mm i.d. fused-silica capillary column with a chemically bonded 0.25-μm DB 5-MS UI stationary phase (J&W Scientific, Folsom, CA). The injector temperature was 260°C, the purge flow was 20 mL/min, and the purge was turned on after 75 s. The gas flow rate through the column was 1 mL/min, and the column temperature was held at 70°C for 2 min, then increased by 20°C/min to 320°C, and held there for 4 minutes. Helium was used as carrier gas with a flow rate of 1 ml/min. The column effluent was introduced into the ion source of a Pegasus HT time-of-flight mass spectrometer, GC/TOFMS (Leco Corp., St Joseph, MI). The transfer line and ion source temperatures were 250 and 200°C, respectively. Ions were generated by a 70 eV electron beam at an ionization current of 2.0 mA, and 20 spectra/s were recorded in the mass range 50−800 m/z. The acceleration voltage was turned on after a solvent delay of 290 s and the detector voltage was 1520 V. Samples with methyl stearate in heptane (5ng/ml) were analyzed in addition to the study samples allowing continuous check of instrumental sensitivity. Retention indices were calculated by use of in run alkane series (C8-C40). Data from GC/MS analysis was exported in NetCDF (Network Common Data Form) format and processed in MATLAB 8.1.0 R2013a (Mathworks, Natick, MA, USA).

### Data processing and metabolite identification

An in-house script for MATLAB was used for pre-processing, followed by hierarchical multivariate curve resolution (H-MCR), as previously described [15]. Peak areas of internal standards were calculated with a raw data analysis in-house script (RDA), and used for normalization. Metabolites were identified using resolved spectral window searches in NIST MS Search 2.0 and an in-house spectral library established by Umeå Plant Science Centre and the library at the Max Planck Institute (http://csbdb.mpimp-golm.mpg.de/csbdb/gmd/msri/gmd_msri.html). Positive identification was based on a combination of match values, conformity with high mass peaks and good agreement with retention index. All putative metabolites identified were recalculated with the RDA script using unique m/z features and the resulting peak areas were used in statistical analyses.

### Raw data cleaning and statistical analysis

**Fig 1:**
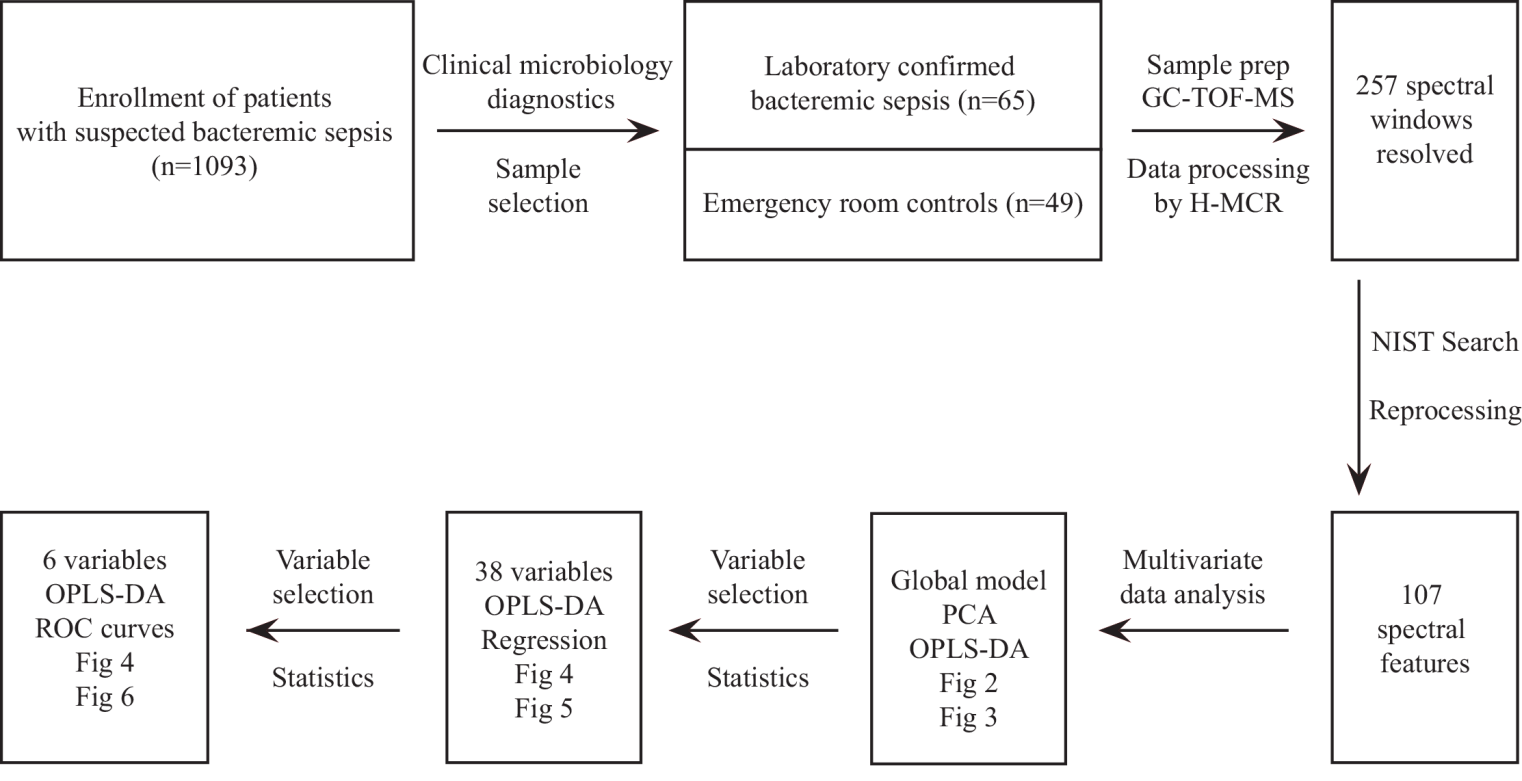
Overview of the study design. The workflow of the study began with the collection of blood samples and ended with the identification and evaluation of 6 metabolite variables for prediction of bacteremic sepsis.

Multivariate data analysis was carried out in the software SIMCA (SIMCA 13.0, Umetrics AB, Umeå, Sweden). Pooled quality control samples were used for quality assurance. Prior to multivariate modeling, the data set was divided into a work set (n = 72) and a test set (n = 42) (**Fig 1**). Work set samples were used for modeling and test set samples for validation of the models. The raw data were mean centered, unit variance scaled, and log-transformed. Principal component analysis (PCA) was used for obtaining an overview of the data and detecting outliers. Biological replicates, technical replicates and quality control samples were used for analysis of skewness and for determination of reproducibility. To clean the raw data, metabolite features with high positive or negative skewness after unit variance scaling and log-transformation as well as features identified with a relative standard deviation of >50% among quality control samples were excluded. All remaining resolved spectral windows were used in orthogonal partial least squares discriminant analysis (OPLS-DA) where metabolites with the strongest contribution to class separation were identified. [16].

Significance testing of the OPLS-DA models was performed with cross validated ANOVA (CV-ANOVA) and the predictive power was evaluated using the test set samples. Additional cross validation including estimates of the number of components and *P*-values for group separations was performed using CV-ANOVA.

Demographic, clinical and laboratory data were analyzed with the independent t-test, Mann-Whitney or Fischer’s exact test. The raw peak areas of metabolites identified with multivariate data analysis were further analyzed with MANOVA, and the Mann-Whitney U test. Binary logistic regression was used to model the response variable bacteremic sepsis (taking the value 1) or ER control (taking the value 0) with the continuous peak areas of metabolites as explanatory variables. Using work set sample data only, suitable regression models and combination of explanatory variables were determined by entering different metabolite combinations. All the binary regression models selected were validated using test set samples resulting in a probability of each sample to belong into the categories bacteremic sepsis (1) or ER control (0). A probability of >0.5 was deduced as bacteremic sepsis and a probability of <0.5 as ER control. An analogous binary regression modelling and validation procedure was performed using the standard clinical measurements (clinical biochemistry and physiology values). The predictive performance was further evaluated by entering the binary data in 2x2 tables for calculation of accuracy, sensitivity and specificity with 95% confidence interval (CI) using Fischer’s exact test. Receiver operator characteristics (ROC) were also employed for evaluation of diagnostic performance. The analyses were executed in SPSS 21.0 (IBM Statistics SPSS, New York, US) and GraphPad Prism 6.00, (San Diego California US). Tests were considered significant at *P*< .05.

## Results

### Patient and sample characteristics

An overview of patient demographics, laboratory measurements, clinical measurements, co-morbidities, and the study workflow are shown in **Table 1** and **Fig 1**. Sixty-five patients with bacteremic sepsis had significantly higher C-reactive protein, white blood cell counts, and longer hospital stay than 49 ER controls. Eighty percent of patients with bacteremic sepsis and 45% of ER controls fulfilled at least two SIRS criteria (*P* < .001). There was no statistically significant difference in gender distribution, age, or co-morbidities between patients with bacteremic sepsis and ER controls (**Table 1**) or between work set and test set data as a whole (not shown). Frequencies of the different bacterial causes of bacteremia among 42 patients in the work set was 0.36 for *E*. *coli* (n = 15), 0.21 for *S*. *pneumoniae* (n = 9), 0.19 for *S*. *pyogenes* (n = 8), 0.19 for *S*. *aureus* (n = 8), and 0.05 for *Klebsiella pneumoniae* (n = 2). Frequencies among 23 patients in the test set was 0.43 for *E*. *coli* (n = 10), 0.13 for *S*. *pneumoniae* (n = 3), 0.13 for *S*. *pyogenes* (n = 3), 0.13 for *S*. *aureus* (n = 3), and 0.17 for *Klebsiella pneumoniae* (n = 4).

**Table 1:** Characteristics and clinical variables of patients.

		Work set		Test set		
Variable (no. analyzed)^a^		42 with bacteremic sepsis	30 ER controls	23 with bacteremic sepsis	19 ER controls	*P* Value for difference, all bacteremic sepsis cases versus all ER controls
Patient characteristics						
	Age in y (114)	71 ± 17	68 ± 17	71 ± 14	67 ± 19	.850
	Percent males (114)	52	50	57	53	.768
	No. with diabetes (113)	8	4	3	5	.999
	No. with cardiovascular disease (114)	11	8	10	4	.409
	No. with malignancy (111)	4	5	2	3	.255
	No. with COPD (110)	3	4	4	6	.185
Clinical parameters							
	Temperature in °C (108)	39.0 ± 1.1	37.9 ± 0.7	38.6 ± 0.9	38 ± 1	< .001
	Systolic blood pressure in mmHg (104)	133 ± 29	142± 26	125 ± 29	143 ± 32	.051
	Respiration rate per minute (91)	23 ± 8	22 ± 7	26 ± 13	22 ± 6	.690
	Percent with SIRS ≥ 2 (102)	80	42	80	50	< .001
	No. with severe sepsis (107))	9	0	9	0	< .001
	No. dead within 30 days (112)	5	1	1	0	.398
	MEDS score^b^ (108)	3.2 ± 4.0	2.6 ± 2.2	4.7 ± 3.9	3.0 ± 2.9	.083
	MEWS score (107)	2.5 ± 3.9	2.0 ± 2.0	3.6 ± 2.7	2.4 ± 2.3	< .001
	CRB-65 score (107)	1.1 ± 0.9	0.8 ± 0.5	1.2 ± 0.9	0.7 ± 0.6	.064
	Charlson score (112)	1.3 ± 1.3	1.5 ± 1.7	2.0 ± 1.9	1.5 ± 1.8	.641
	Days in hospital (112)	12 ± 14	4 ± 4	7 ± 6	4 ± 2	.002
	Days in Intensive Care (112)	4	1	2	0	.134
Clinical Chemistry							
	C-reactive protein in mg/L (114)	175 ± 128	50. ± 54	157 ± 112	79 ± 74	< .001
	Hemoglobin concentration in g/L (114)	125 ± 16	132 ± 19	120 ± 14	130 ± 17	.027
	White blood cell concentration ×10^9^/L (114)	14 ± 5	9 ± 4	16 ± 10	9 ± 3	< .001
	Thrombocyte concentration ×10^9^/L (114)	222 ± 90	274 ± 91	245 ± 106	260 ± 83	.023
	Creatinine in μmol/L (114)	107 ± 45	78 ± 25	95 ± 49	93 ± 49	.022

^a^ Data are presented as mean with standard deviations.

^b^ MEDS, mortality in emergency department sepsis; MEWS, modified early warning score; CRB-65, pneumonia severity score.

### Global metabolomic analysis of bacteremic sepsis

**Fig 2:**
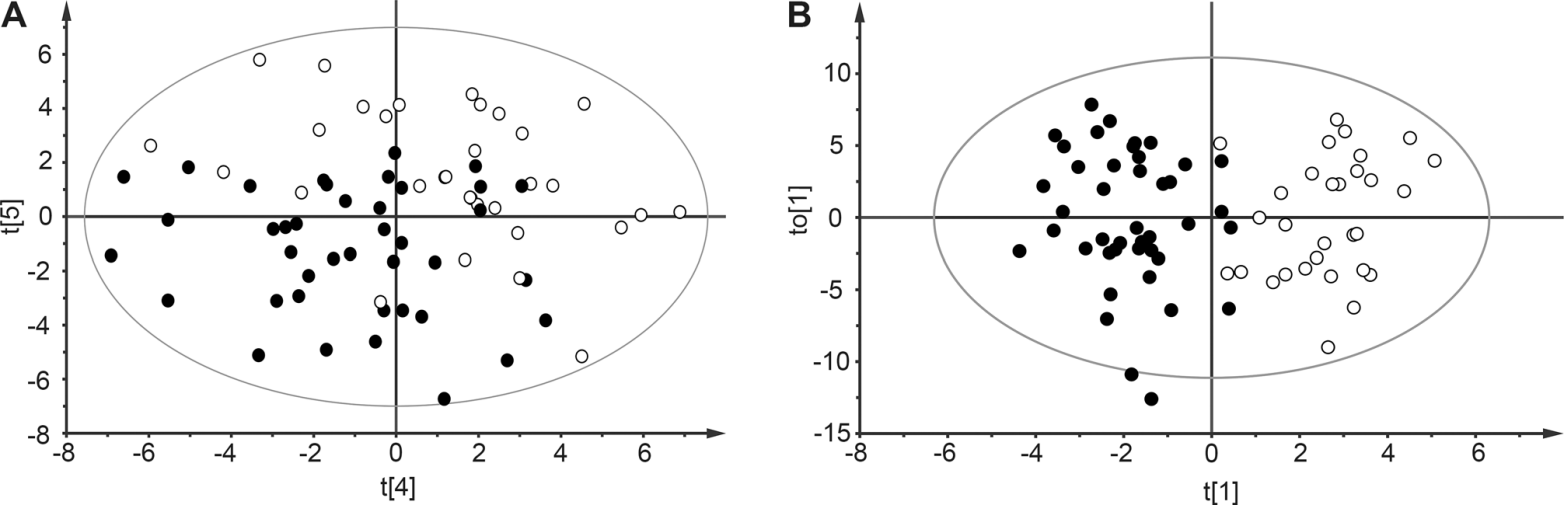
Multivariate data analysis with work set samples (n = 72) using 107 metabolites. Panel A shows a PCA plot of the 4/5th principal component separating bacteremic sepsis (black circles) from ER controls (white circles). Panel B shows an OPLS-DA plot of t1/t(o)1, discriminating bacteremic sepsis (black circles) from ER controls (white circles), (*P* = 4.8×10^−12^).

**Fig 3:**
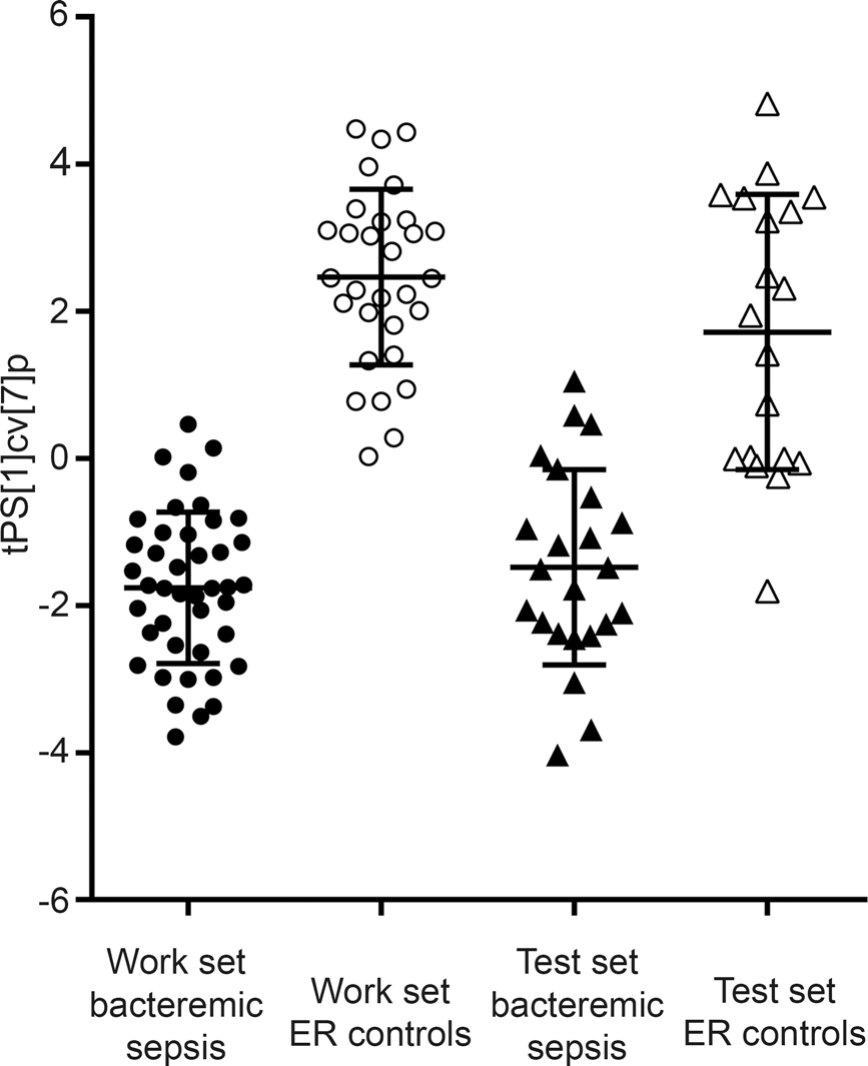
Scatter plot of class discrimination based on cross-validated scores in an OPLS-DA model using 107 metabolites. The samples of the work set with bacteremic sepsis (black circles) and the work set of ER controls (open circles) were used for modeling. Validation using the samples of the test set with bacteremic sepsis (black triangles) and the test set of ER control (open triangles) is shown. The Y-axis represents the seven fold cross-validated predictive score vector 1. Error bars represent mean score values with 95% confidence intervals.

Via hierarchical multivariate curve resolution of GC-MS data obtained from each of the patient samples 254 spectral windows were resolved, each representing a putative metabolite. After reprocessing of data with the RDA script and raw data cleaning (see Materials and Methods), 107 metabolites remained and were used for modeling (detailed in **S1 Table**). Separation of patients with bacteremic sepsis from ER controls was obtained in the fifth score vector by PCA (R2 = 0.762; Q2 = 0.448) (**Fig 2A**). No credible class discrimination among the five different bacterial species causing bacteremic sepsis was obtained by PCA or OPLS-DA. A global OPLS-DA model (R2Y = 0.792; Q2 = 0.621) using the 107 metabolites however successfully discriminated bacteremic sepsis and ER controls (**Fig 2B**). A validation of the OPLS-DA model using the test set metabolite data that had not been used for creating the model verified good model performance (**Fig 3**).

### Subsets of metabolites for prediction of bacteremic sepsis

**Fig 4:**
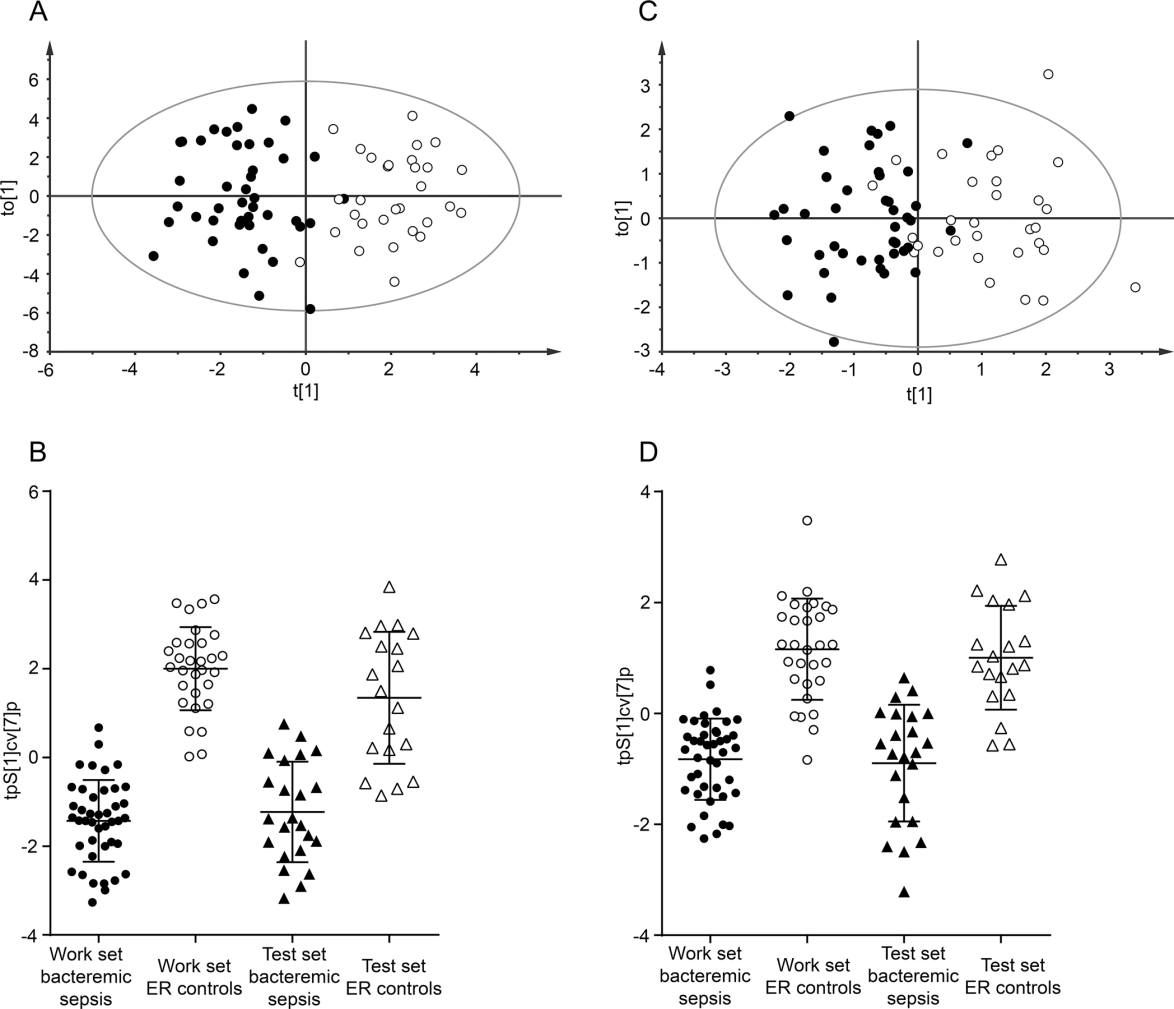
OPLS-DA models using subsets of metabolites and predictions of test set samples. Panel A shows an OPLS-DA plot using work set samples and 38 metabolites discriminating bacteremic sepsis (black circles) from ER controls (open circles), (*P* = 2.0×10^−17^). Panel B shows a scatter plot of the class discrimination using cross-validated scores (tPS[1]cv[7]p) of an OPLS-DA model with 38 metabolites. Work set bacteremic sepsis (black circles), work set ER controls (open circles), test set bacteremic sepsis (black triangles) and tests set ER control (open triangles) are shown. Panel C shows an OPLS-DA plot using work set samples and 6 metabolites discriminating bacteremic sepsis (black circles) from ER controls (open circles), (*P* = 4.1×10^−11^). Panel D shows a scatter plot of the class discrimination using cross-validated scores (tPS[1]cv[7]p) of an OPLS-DA model with 6 metabolites. Work set bacteremic sepsis (black circles), work set ER controls (open circles), test set bacteremic sepsis (black triangles) and tests set ER control (open triangles) are shown. Error bars in panel B and D represent mean score values with 95% confidence intervals.

**Fig 5:**
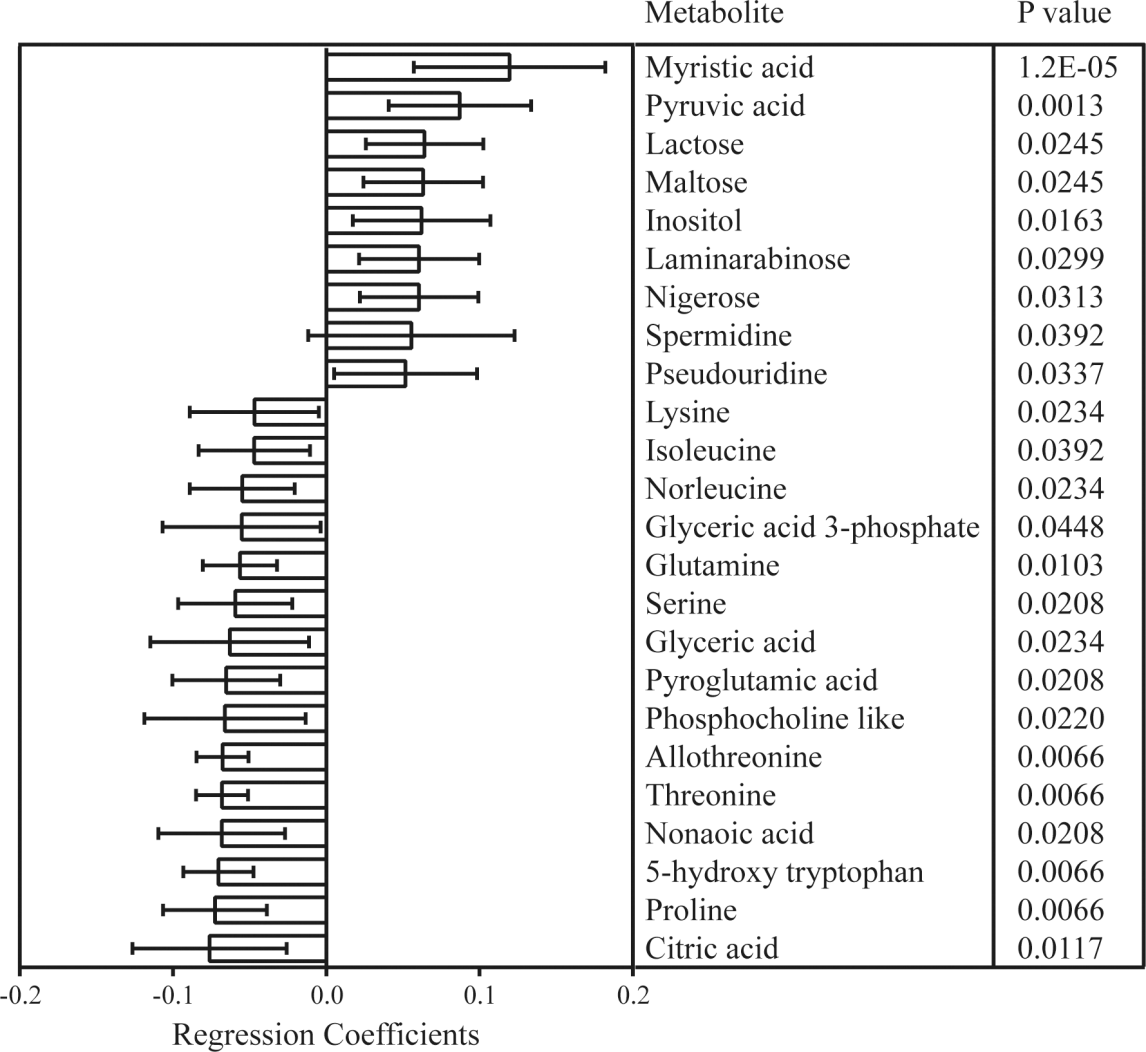
The regression coefficient plot for the OPLS-DA model with 38 metabolites using work set samples. Positive regression coefficients indicate a positive correlation with bacteremic sepsis and negative coefficient a negative correlation.

After exclusion of variables with little contribution to discrimination, a new OPLS-DA model for work set samples using 38 metabolites was fitted with good class separation (R2X = 0.766; Q2 = 0.712) **Fig 4A**. Model validation using test set samples resulted in correct classification (**Fig 4B)**. Statistical testing by Mann-Whitney U and false discovery rate correction identified 24 metabolites to be significantly altered between the classes (**Fig 5**). By further analysis of the variable importance plot and the loading plot alongside logistic regression, 6 metabolites with the strongest contribution to class separation were identified. An OPLS-DA model with these 6 metabolites was fitted (R2X = 0.71; Q2 = 0.66), again with good class discrimination **(Fig 4C)** and ability to predict bacteremic sepsis using test samples (**Fig 4D)**.

### Metabolite based classification models versus conventional diagnostics

**Fig 6:**
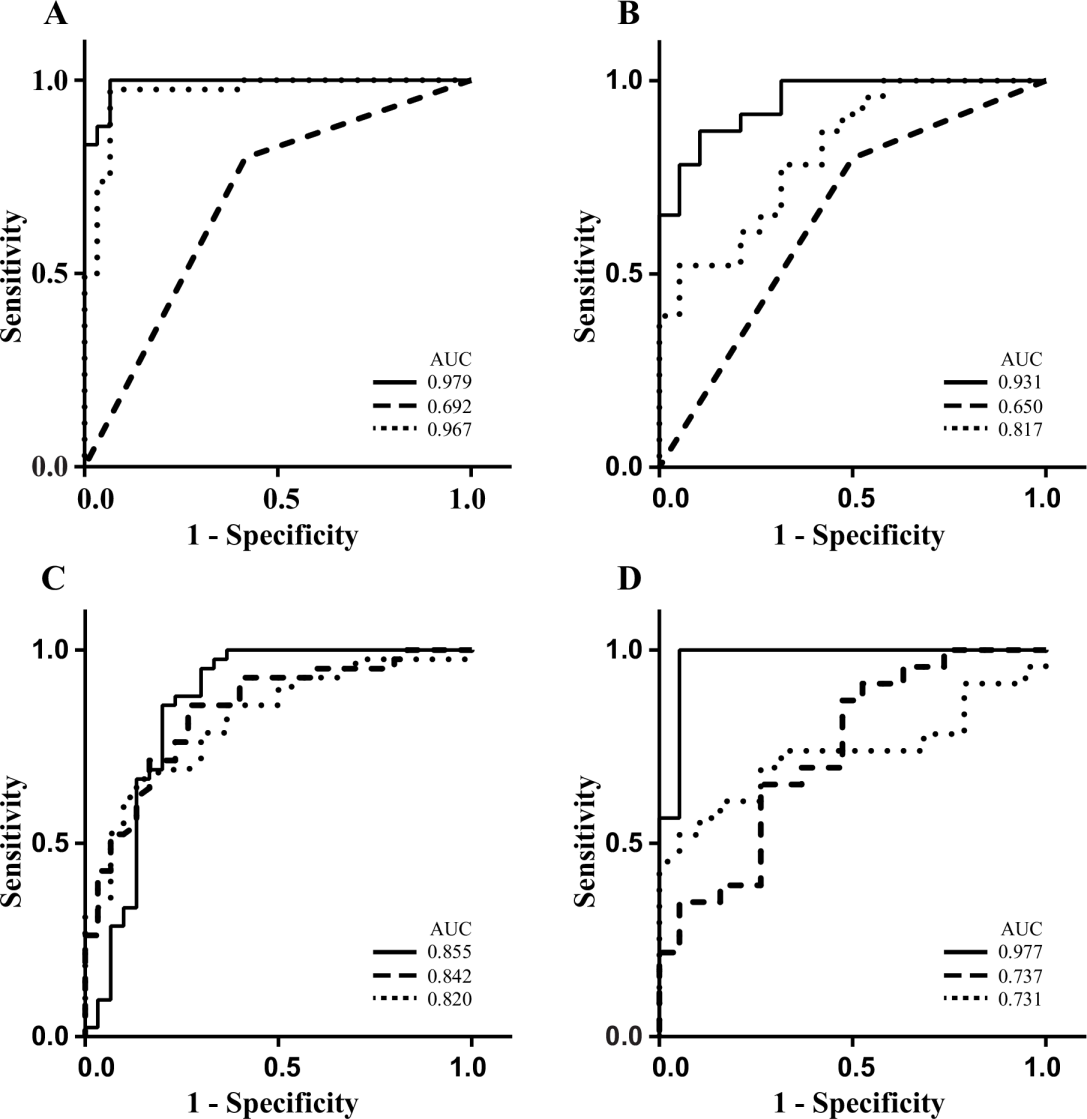
ROC curves of metabolites and laboratory diagnostic variables available in the clinic for the prediction of bacteremic sepsis. Panel A shows logistic regression modelling on work set samples using 6 metabolites (solid line), the combination of temperature, C-reactive protein, thrombocyte, and white blood cell count (dotted line) and the combination score SIRS ≥2 (dashed line). Panel B shows prediction on test set samples using 6 metabolites (solid line), the 4 best clinical variables (dotted line) and the combination variable SIRS ≥2 (dashed line). Panel C shows logistic regression modelling on single variables of work set samples for myristic acid (solid line), white blood cell count (dotted line) and C-reactive protein (dashed line). Panel D shows prediction on test set samples using myristic acid (solid line), white blood cell count (dotted line) and C-reactive protein (dashed line).

The 6 metabolites with the strongest ability to separate bacteremic sepsis from ER-controls were annotated as myristic acid, citric acid, isoleucine, norleucine, pyruvic acid and a phosphocholine like derivative. A binary logistic regression model using the 6 metabolite data of work set samples for predicting bacteremic sepsis demonstrated a sensitivity of 0.95 (95% CI 0.84–0.99) and a specificity of 0.90 (95% CI 0.82–0.99) with an AUC of 0.98 (95% CI 0.97–1.00). The best binary logistic regression model that could be derived using infection laboratory variables available from the clinic (C-reactive protein, leukocyte and thrombocyte count) combined with measurement of the body temperature displayed a sensitivity of 0.98 (95% CI 0.87–0.99) and a specificity of 0.92 (95% CI 0.74–0.99) with an AUC of 0.97 (95% CI 0.93–1.01) using work set samples (**Fig 6A, Table 2**). Evaluation of the regression models using test set data showed a sensitivity of 0.91(95% CI 0.69–0.99) and a specificity 0.84 (95% CI 0.58–0.94) for 6 metabolites, and sensitivity of 0.83 (95% CI 0.61–0.95) and specificity of 0.56 (95% CI 0.31–0.78) for the 4 best infection variables available from the clinic. (**Fig 6B, Table 2**).

**Table 2:** AUC and model performance for work set and test set.

Performance variables		Six metabolites	4 clinical variables	SIRS score	White blood cell count	C-reactive protein	Myristic acid
**Work set data (n = 72)**^**a**^							
	Accuracy (%)	93.1	95.5	71.9	76.4	76.4	81.8
	Sensitivity	0.952	0.976	0.800	0.857	0.762	0.905
	Specificity	0.900	0.920	0.583	0.633	0.767	0.700
	PPV	0.930	0.953	0.762	0.766	0.821	0.809
	NPV	0.931	0.958	0.636	0.760	0.697	0.840
	AUC	0.979	0.967	0.692	0.820	0.842	0.855
**Test set data (n = 42)**^**a**^							
	Accuracy (%)	88.1	70.7	65.8	69.0	64.3	97.6
	Sensitivity	0.913	0.826	0.800	0.652	0.739	1.000
	Specificity	0.842	0.556	0.500	0.737	0.526	0.947
	PPV	0.875	0.704	0.640	0.750	0.654	0.958
	NPV	0.889	0.714	0.692	0.636	0.625	1.000
	AUC	0.931	0.817	0.650	0.731	0.737	0.977

^a^ Model performances were calculated with Fischer’s exact test using 2x2 tables of predicted probabilities obtained via logistic regression.

The predictive capacity of SIRS scores were evaluated by a regression model using dichotomy SIRS values set at 0–2 or ≥ 2 which resulted in a sensitivity of 0.80 (95% CI 0.64–0.91), a specificity of 0.58 (95% CI 0.37–0.78) and an AUC of 0.69 (95% CI 0.55–0.83) using the work set samples (**Fig 6A, Table 2**).

By logistic regression as well as multivariate modeling, myristic acid was identified as the strongest contributor to class separation among the metabolites. A regression model on work set data using myristic acid alone showed a sensitivity of 0.91(95% CI 0.77–0.97) and a specificity of 0.70 (95% CI 0.50–0.85) with an AUC of 0.86 (95% CI 0.75–0.96) (**Fig 6C**). Regression models on work set data using single variables available from the clinic showed a sensitivity of 0.86 (95% CI 0.71–0.95), and specificity of 0.63 (95% CI 0.44–0.80) for leucocytes and a sensitivity of 0.76 (95% CI 0.60–0.88) and a specificity of 0.77 (95% CI 0.58–0.90) for C-reactive protein (**Table 2**). The three single markers showed similar ROC curves for the work set samples (**Fig 6C**) but with superior predictive power on test set samples for myristic acid (**Table 2, Fig 6D**).

### Estimation of metabolite quantities

**Fig 7:**
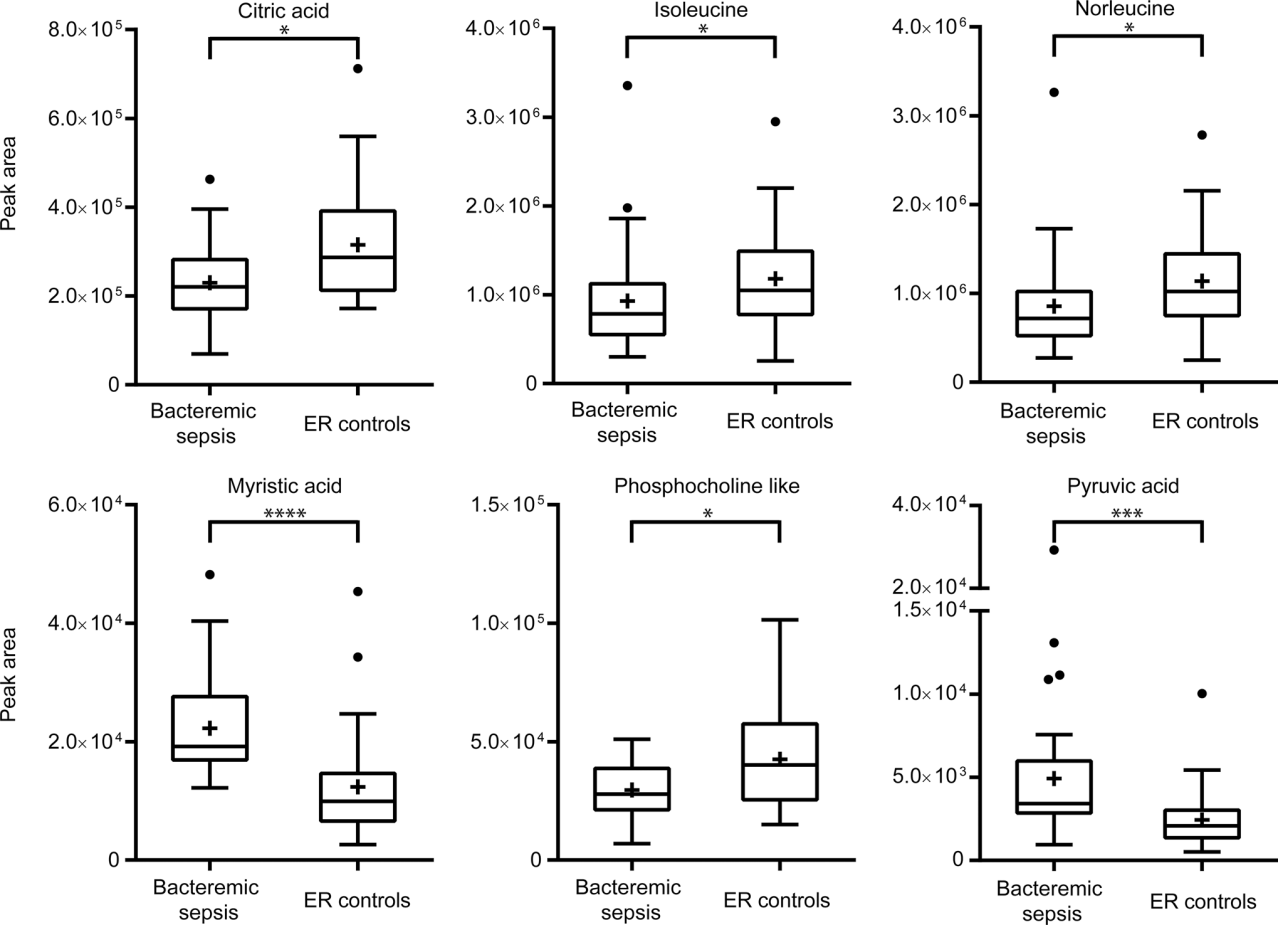
Tukey’s box-and-whisker plots for work set samples using the 6 most important metabolites. Values are corresponding to chromatogram peak areas. Outliers are represented by dots outside the 1.5 interquartile range of the 25 respective 75 percentile. Mean values are indicated by a plus sign. The P values were derived from MannWhitneyU tests *P < .05, **P < .01, ***P < .005, ****P < .001.

Analysis of mass spectrometry peak areas corresponding to myristic acid indicated elevated levels in bacteremia **(Fig 7)**. Similarly, elevated levels of pyruvic acid and two disaccharides were observed. The levels of norleucine, phosphocholine like molecule and citric acid were lower for bacteremic sepsis cases compared to ER controls.

## Discussion

Our study suggests that biomarkers identified by metabolomic analysis of blood taken in the ER can be used for differentiation between patients with and without bacteremic sepsis. New tests of infection to help clinicians in their decisions on early antibiotic treatment and an appropriate level of care are much needed. Early identification of patients with bacteremia is important because these patients have worse outcomes and may need targeted treatment interventions [17, 18]. A negative biomarker test, in contrast, may help a clinician to refrain from the use of unnecessary broad-spectrum antibiotics and direct the treatment and additional diagnostics for other causes of disease.

The concept of using diagnostic patterns, *i*.*e*. using multiple characters as input, is largely unexplored in clinical microbiology diagnostics despite that the use of patterns rather than individual characters is well established in several clinical scoring systems for various medical conditions, *e*.*g*., in SIRS scores, cancer stage scores, and heart failure scores. The pattern of 6 metabolites identified in this study showed specificity and sensitivity values for bacteremic sepsis that were comparable to a combination of C-reactive protein, leukocyte count, thrombocyte count, and body temperature which was the best possible combination we found among clinically available parameters. Both these patterns provided better predictions of bacteremic sepsis than SIRS scores, which have been suggested to be helpful in deciding the need for performing blood cultures [19, 20]. We believe that the interpretation of patterns is a feasible and helpful future approach for diagnosis of infectious conditions that can be adapted in the clinic by for example using apps in mobile devices. Similar functions using multiple characters are already in clinical use for various clinical scores [21].

Among the 6 metabolites useful for discriminating bacteremia from ER controls especially one of them, myristic acid, identified bacteremic sepsis with higher accuracy than the C-reactive protein which is considered one of the better acute-phase markers available for clinical management and decisions on the need for antibiotic therapy [22]. Our study does not reveal the physiological role of myristic acid (one of several saturated short and medium chain fatty acids present in the human metabolome). Such compounds, however, have been implicated to play a role in the inflammatory cascade through cytokine release from monocytes and macrophages. An increased expression of cyclooxygenase-2 through activation of Toll-like receptors via nuclear factor kappaB has been proposed as the mechanism [23]. An infection response involving fatty acids was also recently described in experimental sepsis of humans. In that experimental model, increased levels of tetradecanedionate, stearate and eicosenoate and other fatty acids were observed with transcriptomic changes pointing at a shift in energy production towards glycolysis and depletion of a number of amino acids; findings which are parallel to the metabolite patterns observed in the current study [24]. Our observations of decreased levels of isoleucine and norleucine as components of the 6 metabolite pattern in bacteremic sepsis are in line with observations made by others of decreased levels of amino acids in sepsis [25, 26]. Importantly, several additional amino acids were found to be down regulated in bacteremic sepsis in the current study, again in accordance with recent studies by others [25, 26]. Furthermore, we noted increased levels of various disaccharides and tri saccharides, possibly resulting from amino acid catabolism during sepsis [27].

Unlike in other metabolomics studies of sepsis, we have used whole blood as the sample matrix. Other studies have used serum or plasma and although our sample choice was mainly based on availability, it has recently been shown that in metabolomics analyses using proton nuclear magnetic resonance spectroscopy whole blood provided more information as compared with serum or plasma [28]. In that study it was suggested that the use of whole blood may be particularly important for studies in diseases such as sepsis in which red blood metabolism is altered. In line with this reasoning it is possible that some of the metabolite patterns described here were released from red blood cells [28–30]. Another difference from previous metabolomics studies of sepsis was that we used a blood sampling protocol that is close to a clinical situation with some variation in handling and storage times, and possibly with ongoing metabolic changes after sampling. We think that to be useful in the clinic, the diagnostic metabolites should be stable to such variations but we acknowledge that we might have missed some information of transient nature.

The design of this study included multiple bacterial causes of bacteremic sepsis because we aimed at finding agent-specific metabolite patterns. In contrast to a previous study on *S*. *aureus* and *E*. *coli* sepsis in the intensive care unit [9], we could not verify such patterns in the current study. The presented results are in agreement with a recent much larger clinical trial identifying no major differences among patients infected with *S*. *pneumoniae*, *S*. *aureus*, or *E*. *coli* [7].

An obvious limitation with the current study is the use of an idealized study design with only two patient categories receiving an unambiguous final medical diagnoses, i.e. cases with laboratory verified bacteremic sepsis and ER controls without bacteremia. In clinical settings there is typically a large group of patients that stays without laboratory confirmation at discharge from hospital and this group was not taken into account in the current study design. It was also evident from the analysis of clinical characteristics of the patients with and without bacteremic sepsis that the group with a subsequent bacteremia diagnosis generally had a more severe disease with higher body temperature and clinical disease severity scores. This group difference in disease severity may be an important explanation to the metabolite patterns observed. There were also some age category differences between groups, which may have had effect on the results. It also remains unclear if the metabolite patterns detected primarily mirrored host responses, was derived from infecting bacteria, or was a combination of these two disease processes.

In conclusion, this study showed that measurement of a small set of metabolites in whole blood collected at admission to hospital could predict bacteremic sepsis. In particular, elevated levels of myristic acid were associated with subsequent positive blood culture. The results are encouraging because they suggest that a metabolomic approach for evaluation of patients suspected with infection can provide new diagnostic tools.

## Supporting Information

S1 Table Features of 107 metabolites in 114 whole blood samples. (XLSX)
